# Exosomes released from human induced pluripotent stem cells-derived MSCs facilitate cutaneous wound healing by promoting collagen synthesis and angiogenesis

**DOI:** 10.1186/s12967-015-0417-0

**Published:** 2015-02-01

**Authors:** Jieyuan Zhang, Junjie Guan, Xin Niu, Guowen Hu, Shangchun Guo, Qing Li, Zongping Xie, Changqing Zhang, Yang Wang

**Affiliations:** Department of Orthopedic Surgery, Shanghai Jiao Tong University Affiliated Sixth People’s Hospital, Shanghai, China; Institute of Microsurgery on Extremities, Shanghai Jiao Tong University Affiliated Sixth People’s Hospital, Shanghai, China; Graduate School of Nanchang University, Nanchang, Jiangxi China

**Keywords:** Induced pluripotent stem cells (iPSCs), Exosomes, Wound healing, Angiogenesis

## Abstract

**Background:**

Human induced pluripotent stem cell-derived mesenchymal stem cells (hiPSC-MSCs) have emerged as a promising alternative for stem cell transplantation therapy. Exosomes derived from mesenchymal stem cells (MSC-Exos) can play important roles in repairing injured tissues. However, to date, no reports have demonstrated the use of hiPSC-MSC-Exos in cutaneous wound healing, and little is known regarding their underlying mechanisms in tissue repair.

**Methods:**

hiPSC-MSC-Exos were injected subcutaneously around wound sites in a rat model and the efficacy of hiPSC-MSC-Exos was assessed by measuring wound closure areas, by histological and immunofluorescence examinations. We also evaluated the in vitro effects of hiPSC-MSC-Exos on both the proliferation and migration of human dermal fibroblasts and human umbilical vein endothelial cells (HUVECs) by cell-counting and scratch assays, respectively. The effects of exosomes on fibroblast collagen and elastin secretion were studied in enzyme-linked immunosorbent assays and quantitative reverse-transcriptase–polymerase chain reaction (qRT-PCR). In vitro capillary network formation was determined in tube-formation assays.

**Results:**

Transplanting hiPSC-MSC-Exos to wound sites resulted in accelerated re-epithelialization, reduced scar widths, and the promotion of collagen maturity. Moreover, hiPSC-MSC-Exos not only promoted the generation of newly formed vessels, but also accelerated their maturation in wound sites. We found that hiPSC-MSC-Exos stimulated the proliferation and migration of human dermal fibroblasts and HUVECs in a dose-dependent manner in vitro. Similarly, Type I, III collagen and elastin secretion and mRNA expression by fibroblasts and tube formation by HUVECs were also increased with increasing hiPSC-MSC-Exos concentrations.

**Conclusions:**

Our findings suggest that hiPSC-MSC-Exos can facilitate cutaneous wound healing by promoting collagen synthesis and angiogenesis. These data provide the first evidence for the potential of hiPSC-MSC-Exos in treating cutaneous wounds.

## Introduction

The skin is frequently injured by acute and chronic wounds, such as diabetic skin ulcerations or extensive burns, which cause physical and mental suffering in affected individuals, as well as heavy financial burdens at the familial and societal levels. Although numerous investigations have focused on expediting the wound healing process, definitive treatments are currently unavailable.

In recent years, advances in stem cell transplantation therapy have shown promise in promoting skin-wound healing. Mesenchymal stem cells (MSCs) and embryonic stem cells (ESCs) have been most commonly studied, and they have been effective in promoting wound healing when injected into wound sites, alone or in combination with biological materials [1-3].

However, the procedures involved in harvesting MSCs from adult tissues are invasive and the proliferation and differentiation capacities of MSCs are limited after several passages in culture. Moreover, the proliferation and differentiation potential of MSCs declines significantly when age-related disorders occur and with increasing age [4,5]. The application of ESCs faces substantial ethical and safety hurdles. These factors restrict the clinical application of MSCs and ESCs. Induced pluripotent stem cells (iPSCs) are similar to ESCs in terms of morphology, self-renewal, and differentiation capacity [6,7], and iPSCs can be generated from any tissue type in the body. While iPSCs show unlimited growth capacity, are not associated with ethical concerns, are superior to traditional MSCs and ESCs, and can serve as an inexhaustible source for stem cell transplantation therapy, but iPSCs are also potentially tumorigenic [8]. Mesenchymal stem cells derived from iPSCs (iPSC-MSCs) offer the advantages of both MSCs and IPSCs. Specifically, abundant MSCs can be generated from iPSCs, and while iPSC-MSCs can be passaged >40 times in culture and sustain the self-renewal capacity characteristic of MSCs [9], they are also no longer tumorigenic [10,11]. Patient-specific iPSC-MSCs can be used for autologous transplantation without immunological rejection and the beneficial effects of iPSC-MSCs in tissue repair have already been demonstrated [9,12,13]. Despite their apparent advantages, issues of allogeneic and xenogeneic immunological rejection and chromosomal variation in cell transplantation therapy still exist [14,15]. Hence, the focus of this study was on developing a strategy to overcome the disadvantages of cell transplantation therapy.

Recent studies have demonstrated that stem cell transplantation therapy promotes wound healing mainly through a paracrine mechanism [16-18], and that exosomes play a major role in this mechanism [19-22]. Exosomes–positive for CD9, CD63, CD81, are nano-sized extracellular vesicles (30–100 nm in diameter) generated from many cell types and tissues. Exosomes, contain proteins, mRNAs, and miRNAs [23,24], and are formed within endosomal compartments and released into the extracellular milieu, where then play an important role in intercellular communication [25]. Studies have also indicated that exosomes derived from MSCs (MSCs-Exos) have promise in tissue repair therapy [26-28]. Because iPSC-MSCs exert stronger therapeutic effects in tissue repair than do BMSCs [9,29], we hypothesized that iPSC-MSC-Exos may also promote tissue repair during wound healing.

In the present study, we verified the therapeutic effects of hiPSC-MSC-Exos in cutaneous wound healing. Consistent with our hypothesis, we found that hiPSC-MSC-Exos significantly enhanced wound healing, collagen synthesis, and the genesis of newly formed vessels and mature vessels in wound sites. Our in vitro studies showed that hiPSC-MSC-Exos can promote the proliferation and migration of human fibroblasts and HUVECs, enhance fibroblasts collagen and elastin secretion, and increase tube formation by HUVECs. These data show for the first time that hiPSC-MSC-Exos facilitate cutaneous wound healing by promoting collagen synthesis and angiogenesis.

## Methods

### Derivation and characterization of hiPSC-MSCs

Three human iPSC cell lines were used to generate MSCs. The first human iPSCs line (iPS-S-01) was provided by the Institute of Biochemistry and Cell Biology of the Chinese Academy of Sciences, with permission from Liao and Xiao [30]. Two additional iPSC lines, namely iPSCs-(C1P33) and iPSCs-(PCKDSF001C1), were provided by the South China Institute for Stem Cell Biology and Regenerative Medicine Group of the Chinese Academy of Sciences, with permission from Professor Pei [31]. After 5 days in culture, the mTESR1 (Stemcell) was replaced with MSC medium, prepared by supplementing Dulbecco’s Modified Eagle Medium with 10% FBS, 1% penicillin/streptomycin, 2 mM L-glutamine, and 0.1 mM non-essential amino acids (all supplements from Gibco), and the medium was subsequently changed every 2 days [32]. At 2 weeks cultured in MSC medium, cells were harvested and expanded in 0.1% gelatin-coated dishes with MSC medium. Cells were continually passaged every 5–7 days until they developed a homogeneous fibroblastic morphology, after which they were evaluated in terms of MSC phenotype characteristics and differentiation potentials.

Surface antigens of hiPSC-MSCs were analysed by flow cytometry. Cells at Passage 4 were harvested using trypsin-EDTA (Invitrogen) and incubated for 30 min with 3% Bovine Serum Albumin (BSA, Gibco) in PBS to block nonspecific antigen binding. Cells were next incubated with antibodies (Becton Dickinson) recognizing characteristic human MSC surface markers, including: phycoerythrin (PE)-conjugated anti-CD29, PE-conjugated anti-CD73, fluorescein isothiocyanate-conjugated anti-CD90, allophycocyanin-conjugated anti-CD34, fluorescein isothiocyanate-conjugated anti-CD45, and PE-conjugated HLA-DR. Surface antigens were analysed using a Guava easyCyte™ flow cytometer.

The functional differentiation of hiPSC-MSCs into osteogenic, chondrogenic, and adipogenic lineages was tested in specific culture media. To study osteogenesis, hiPSC-MSCs were incubated in osteogenic medium (Gibco) for 3 weeks and then fixed in 4% paraformaldehyde for 30 min at room temperature (RT). Calcified matrix deposition was detected by Alizarin Red staining. Chondrogenesis was studied by seeding hiPSC-MSCs in chondrogenic medium (Gibco) and maintaining them for 4 weeks in culture. The cells were then fixed in 4% paraformaldehyde for 30 min at RT, and proteoglycans were detected by Alcian Blue staining. For adipogenesis studies, hiPSC-MSCs were incubated in adipogenic medium (Gibco) for 2 weeks, fixed in 4% paraformaldehyde for 30 min at RT, and lipid vacuoles were detected by Oil Red O staining. Tri-lineage differentiation potential was confirmed by qRT-PCR measurement of gene expression levels of markers associated with osteo-, chondro-, and adipogenic differentiation after 7 days in culture with osteo-, chondro-, and adipogenic mediun. The total RNA was isolated using Trizol (Invitrogen), and 1 μg of RNA in a final reaction volume of 20 μl was then reversed-transcribed into complementary DNA (cDNA) using the PrimeScript 1st Strand cDNA Synthesis kit (TaKaRa) according to the manufacturer’s instructions. qRT-PCR was performed using SYBR Premix Ex Taq (Takara) in combination with an ABI 7500 Real-Time PCR System (Applied Biosystems). Threshold cycles of primer probes were normalized to the housekeeping gene GAPDH and translated to relative values. The primers are synthesized as follows: OCN: forward, 5′-CCCCCTCTAGCCTAGGACC-3′, and reverse, 5′-ACCAGGTAATGCCAGTTTGC-3′; SOX 9: forward, 5′-AGCGCCCCCACTTTTGCTCT-3′, and reverse, 5′-GCTCGCCCTTGGGGAACGTG-3′; LPL: forward, 5′-TGGAGGTACTTTTCAGCCAGGAT-3′, and reverse, 5′-CGTGGGAGCACTTCACTAGCT-3′; GAPDH: forward, 5′-ATCCCATCACCATCTTCC-3′, and reverse, 5′-GAGTCCTTCCACGATACCA-3′.

### Isolation and identification of hiPSC-MSC-Exos

After hiPSC-MSCs reached ~80% confluency, the culture medium was replaced with MesenGro hMSC medium (StemRD), and the cells were cultured for an additional 48 h. Conditioned medium (CM) was collected and exosomes were isolated as described previously [33,34]. Briefly, the CM was centrifuged sequentially at 300 × *g* for 10 min and then at 2000 × *g* for 10 min. Next, the cellular debris in CM supernatants was removed by 0.22-μm filtration, and supernatants were ultracentrifuged at 100,000 × *g* for 2 h. Pelleted exosomes were resuspended in PBS, centrifuged at 4000 × *g* until the volume in the upper compartment was reduced to approximately 200 μL. The total protein concentration in exosomes was quantitated using the Micro Bicinchoninic Acid (BCA) Protein Assay Kit (Pierce), according to the manufacturer’s recommended protocol. All procedures were performed at 4°C. Exosome morphologies were observed using a Hitachi H-7650 transmission electron microscope (TEM; Hitachi). Antibodies against the CD9 (1:500; Bioworld), CD63 (1:1000; Bioworld), and CD81 (1:1000; Epitomics) proteins were used to analyse the incorporation of each protein into exosomes in western blots.

### Rat skin wound model and treatment

All procedures were approved by the Animal Research Committee of the Sixth People’s Hospital at the Shanghai Jiao Tong University. Adult male SD rats weighing 250–300 g were used in this study. These rats were anesthetized by intraperitoneal injection of 50 mg/kg pentobarbital. After shaving the rats, 3 wounds (18 mm in diameter) were created on the dorsal skin. Rats were randomly assigned to 3 different treatment groups, which were subcutaneously injected at wound sites with PBS (untreated group), MesenGro hMSC medium (control group), or hiPSC-MSC-Exos (experimental group). Rats in the respective groups were injected with 160 μL of PBS, MesenGro hMSC medium, or hiPSC-MSC-Exos (160 μg) in PBS around the wounds at 4 injection sites, and 40 μL of matching solution (PBS, medium, or exosomes (40 μg)) was applied to the wound beds of the respective groups. Six wounds per treatment were studied by histopathological analysis at Day 7 and 14 post-wounding. Wound-size reduction was calculated using the equation: wound-size reduction (%) = (A_O_ – A_t_)/A_O_ × 100, where A_O_ is the initial wound area, and A_t_ is the wound area at Day 7 or 14 post-wounding.

### Histology

For histological analyses, the excised skin from wound sites was fixed in 10% formalin, dehydrated with a graded-alcohol series, embedded in paraffin, and sectioned perpendicularly to the wound surface into 4-μm-thick sections. Hematoxylin and eosin (H&E) staining was used for histological observations. The percentage of re-epithelisation (E%) was calculated using the equation: E% = W_N_/W_O_ × 100, where W_O_ is the original wound area and W_N_ is the length of newly generated epithelium across the surface of the wound. Masson’s trichrome staining was used to determine the degree of collagen maturity.

### Immunofluorescence study

CD31 and alpha smooth muscle actin (α-SMA) were detected by immunofluorescence to study exosome-induced angiogenesis during the wound healing process. For immunofluorescence staining, excised skin from the wound sites was fixed in 4% paraformaldehyde, dehydrated in 30% sucrose solution, embedded in OCT, and sectioned perpendicularly to the wound surface into 4-μm-thick sections. Tissue sections were blocked in 1% BSA for 30 min at RT, incubated with rabbit anti-CD31 (1:100, Abcam) and mouse anti-α-SMA (1:50, Abcam) antibodies overnight at 4°C. Subsequently, tissue sections were stained with secondary Alexa-Fluor 594-conjugated goat anti-rabbit and Alexa-Fluor 488-conjugated goat anti-mouse antibodies (1:200) and counterstained with DAPI. Images were acquired with an Olympus IX81 microscope. The newly formed vessels were indicated by CD31 positive staining, mature vessels were detected as CD31 and α-SMA double-positive vascular structures. The number of newly formed vessels and mature vessels were determined by counting in five random fields per section between wound edges using Image-Pro Plus 6.

### Treatment of fibroblasts with hiPSC-MSC-Exos in vitro

Human fibroblasts were obtained from 4 donors with written informed consent. The effects of hiPSC-MSC-Exos on the proliferation of human fibroblasts (passage 6–9) were evaluated using the Cell Counting Kit-8 (CCK-8) (Dojindo) according to manufacturer's instructions. Fibroblasts were seeded into 96-well plates at 5 × 10^3^ cells/well. After 12 h, 0, 50, or 100 μg/mL exosomes was added to the wells. The medium was changed daily for 5 days, using fresh medium containing the same exosomes concentrations. Cell proliferation curves were constructed by measuring amount of formazan dye generated by cellular dehydrogenase activity with a microplate reader at a wavelength of 450 nm.

The effects of exosomes on fibroblasts migration were evaluated in scratch assays, as described previously [35]. Briefly, 2 × 10^5^ cells/well were seeded into 6-well plates and incubated for 6 h. Then, the confluent layer of cells was scratched using a sterile 20–200 μl pipette tip. After washing the cells with PBS, 0, 50, or 100 μg/mL exosomes was added. Images were recorded at 0, 12, and 24 h after the monolayers were scratched. Scratched areas were measured using the Image-Pro Plus 6.0 software. The effects of exosomes on fibroblasts migration were evaluated in cell responses level by western blot measurement of fibronectin (Santa Cruz Biotechnology) protein levels at 24 h. The total cellular proteins were first extracted and the cell lysates were cleared by centrifugation at 4°C and 12,000 rpm for 15 min. The protein concentrations of the lysates were quantified using a BCA assay Kit. The cell proteins were separated by standard sodium dodecyl sulfate-polyacrylamide gel electrophoresis (SDS-PAGE) and transferred to polyvinylidene difluoride (PVDF) membranes. After incubation in 5% BSA blocking solution for 1 h, the membranes were incubated overnight at 4°C with anti-fibronectin antibody (Abcam). The membranes were then washed three times with PBS-Tween-20 and incubated with horseradish peroxidase (HPR)-conjugated secondary antibodies at 37°C. The immunoreactive bands were visualized using the ECL chemiluminescence reagent (Millipore).

The effects of exosomes on fibroblasts collagen and elastin secretion were evaluated with an enzyme-linked immune sorbent assay (ELISA) kit (Wes tang Bio-tech) and qRT-PCR. Fibroblasts (2 × 10^5^ cells/well) were seeded into 6-well plates, after which 0, 50, or 100 μg/mL exosomes were added. At Days 1, 2, and 3 in culture, the supernatants were collected and Type I, III collagen and elastin levels were detected using the ELISA kit, according to manufacturer's instructions. The mRNA levels of Type I, III collagen and elastin were examined by qRT-PCR. Threshold cycles of primer probes were normalized to the housekeeping gene GAPDH and translated to relative values. The primer is synthesized as follows: Type I collagen: forward, 5′-AGGACAAGAGGCATGTCTGGTT-3′, and reverse, 5′-TTGCAGTGGTAGGTGATGTTCTG-3′; Type III collagen: forward, 5′-TGGATCAGATGGTCTTCCA-3′, and reverse, 5′-TCTCCATAATACGGGGCAA-3′; GAPDH: forward, 5′-ATCCCATCACCATCTTCC-3′, and reverse, 5′-GAGTCCTTCCACGATACCA-3′.

### Treatment of HUVECs with hiPSC-MSC-Exos in vitro

Human umbilical cords were harvested after obtaining informed consent from donors. The effects of exosomes on HUVECs proliferation and migration were evaluated as described above. In vitro capillary-network formation in Matrigel was monitored by performing tube-formation assays. Culture plates (24-well) were pre-coated with Matrigel, and 5 × 10^4^ cells/well were seeded in M200 medium containing 50 or 100 μg/mL of hiPSC-MSC-Exos and cultured for 4, 6, or 18 h. Separate wells of control cells were grown in M200 medium alone. At each time point, the number of total branch points and tubule lengths in 5 randomly chosen fields were quantified.

### Statistical analysis

All data are shown as mean ± standard deviation (SD). Differences between groups were assessed by one-way analysis of variance (ANOVA) with GraphPad Prism software. P values < 0.05 were considered statistically significant.

## Results

### Characterization of hiPSC-MSCs and hiPSC-MSC-Exos

**Figure 1:**
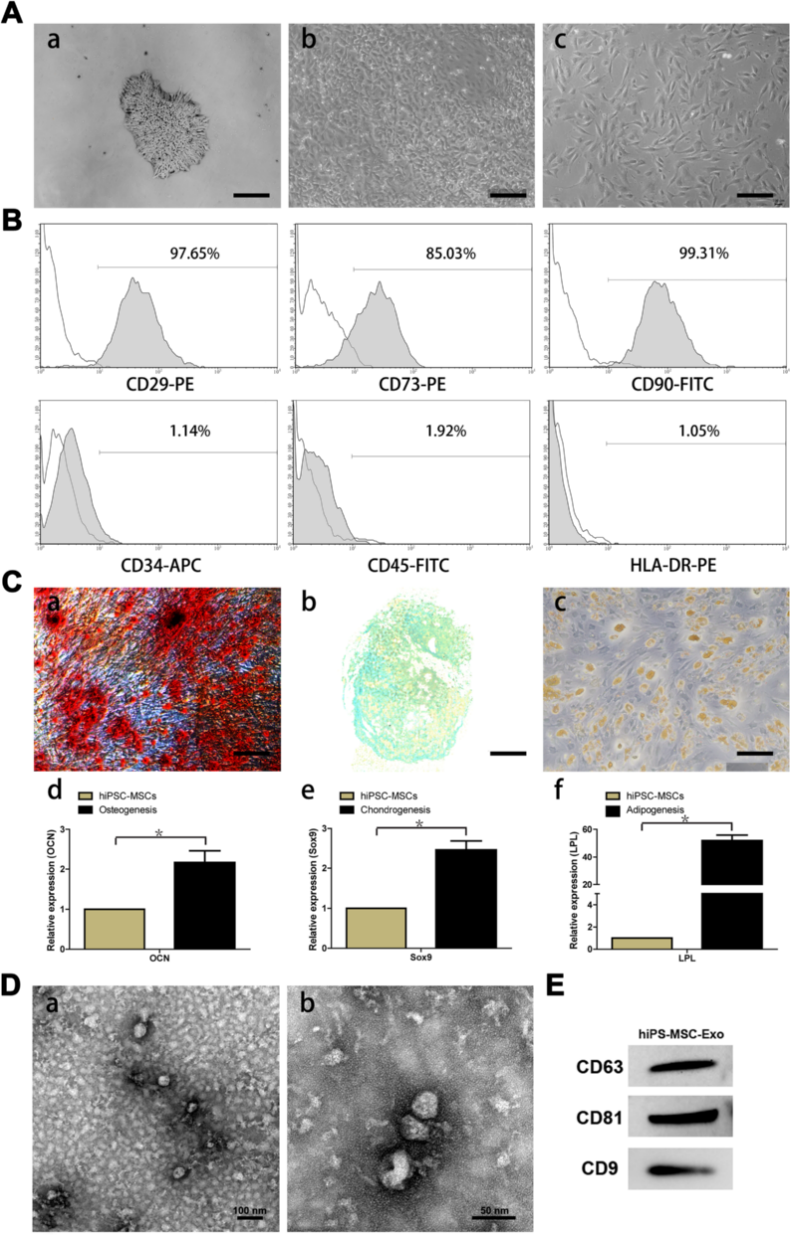
**Characterization of human induced pluripotent stem cell-derived mesenchymal stem cells (hiPSC-MSCs) and hiPSC-MSC-derived exosomes (hiPSC-MSC-Exos). (A)** Light microscopy images demonstrating morphological changes occurring during hiPSCs differentiation into fibroblast-like cells. **(a)** Representative cell morphology of hiPSCs before differentiation. **(b)** Intermediate phase of differentiating the hiPSCs into MSCs. **(c)** Typical fibroblast-like morphology of cells. **(B)** Flow cytometric analysis of the surface markers in hiPSC-MSCs. **(C)** Assessment of the tri-lineage differentiation capacity of iPSC-MSC-like cells. **(a)** Alizarin Red staining for osteocytes after 3 weeks in culture with osteogenic medium. **(b)** Alcian Blue staining for chondrocytes after 4 weeks in culture with chondrogenic medium. **(c)** Oil Red O staining for adipocytes after 2 weeks in culture with adipogenic medium. The qRT-PCR results for OCN **(d)**, Sox9 **(e)**, and LPL **(f)** after 7 days in culture with osteo-, chondro-, and adipogenic mediun. **(D)** Transmission electron microscope images of hiPSC-MSC-Exos morphology. Scale bars = 100 nm and 50 nm, respectively. **(E)** Detection of CD9, CD63, and CD81 incorporation into hiPSC-MSC-Exos by western blotting.

Using a modified one-step induction protocol, we successfully derived human MSCs from 3 different iPSC cell lines. Under the induction conditions used, the hiPSCs showed a tendency to form packed clones with decreased nuclear: cytoplasmic volume ratios, and formed a monolayer with a larger spindle-shaped morphology at the border of the colonies after culture in MSC medium for a few days. After culturing cells on gelatin-coated dishes for 14 days, they were continually passaged when reached to 90% confluence until homogeneous fibroblastic morphologies were observed (Figure 1A). The differentiation of hiPSCs into MSCs was assessed by staining cells with MSC-markers and subsequent analysis by flow cytometry. MSCs were identified as cells positive for CD29, CD73, and CD90 and negative for CD34, CD45, and HLA-DR (Figure 1B).

Tri-lineage MSC differentiation experiments were performed to assess the multipotency of the derived cells. Osteogenesis was studied by measuring the formation of amorphous calcium mineral deposits by Alizarin Red staining after 3 weeks of differentiation (Figure 1Ca). Chondrogenesis was studied by determining the presence of polysaccharides and proteoglycans by Alcian Blue staining after 4 weeks of differentiation (Figure 1Cb). Adipogenesis was studied by measuring the formation of small cytoplasmic lipid droplets by Oil Red O staining after 2 weeks of differentiation (Figure 1Cc). The osteo-, chondro-, and adipogenic differentiation-related genes analysis demonstrated that the gene expression of OCN (Figure 1Cd), Sox9 (Figure 1Ce), and LPL (Figure 1Cf) were upregulated in induced iPSC-MSCs, respectively. These results suggest that the derived hiPSC-MSCs possessed MSC properties and multipotency.

In TEM experiments with hiPSC-MSC-Exos, we observed spheroidal microvesicles that were 30–100 nm in diameter (Figure 1D), indicating the presence of exosomes. Western blotting analyses indicated that the hiPSC-MSC-Exos expressed exosomal markers, such as the CD9, CD63, and CD81 proteins (Figure 1E).

### hiPSC-MSC-Exos promote cutaneous wound healing in rats

**Figure 2:**
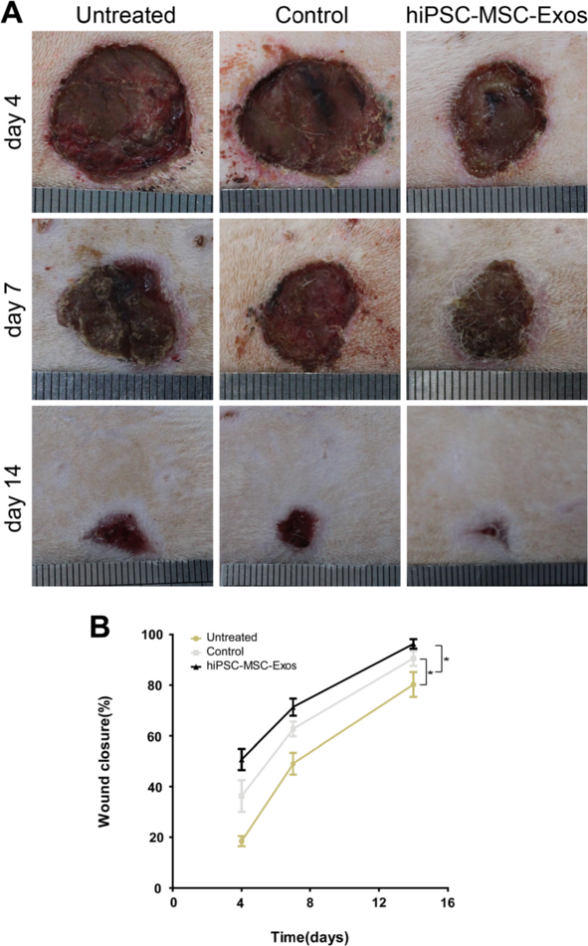
**Rats macroscopic appearances of cutaneous wounds treated with PBS, MesenGro hMSC medium, or hiPSC-MSC-Exos. (A)** Gross view of wounds treated with PBS, MesenGro hMSC medium, or hiPSC-MSC-Exos at 4, 7, and 14 days. **(B)** The effects of treatment with PBS, MesenGro hMSC medium, or hiPSC-MSC-Exos on wound closure at 4, 7, and 14 days. *P < 0.05.

SD rats were anesthetized and 3 wound sites were created in their dorsal skin areas. We evaluated wound healing in 3 groups of animals that were treated with PBS (untreated group), MesenGro hMSC medium (control group), or hiPSC-MSC-Exos (experimental group) in and around the wound sites. Rats treated with hiPSC-MSC-Exos showed greater wound closure than observed in the control and untreated groups at Days 4, 7, and 14 post-wounding (Figure 2A, B).

**Figure 3:**
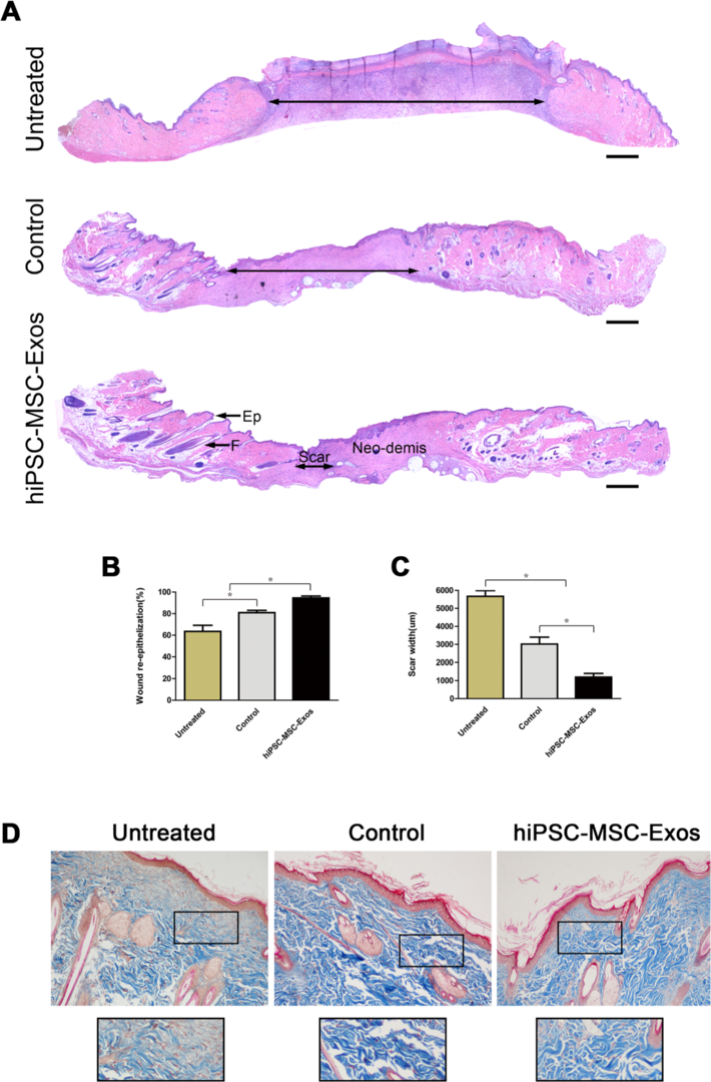
**Rats histological analyses of cutaneous wounds treated with PBS, MesenGro hMSC medium, or hiPSC-MSC-Exos. (A)** H&E staining of wound sections following treatment with PBS, MesenGro hMSC medium, or hiPSC-MSC-Exos at 14 days post-wounding. The double-headed arrows indicate the edges of the scar. The effects of PBS, MesenGro hMSC medium, or hiPSC-MSC-Exos on wound re-epithelialization **(B)** and scar widths **(C)** at 14 days post-wounding. **(D)** Evaluation of collagen maturity by Masson’s trichrome staining of wounds following treatment with PBS, MesenGro hMSC medium, or hiPSC-MSC-Exos at 14 days post-wounding. Scale bar = 500 μm. *P < 0.05; Ep, Epithelium; F, Follicle.

Reduced scar widths and increased collagen maturity are parameters used to assess the degree of wound healing. As shown in Figure 3A–D, hiPSC-MSC-Exos treatment significantly enhanced re-epithelialization compared to that observed in the control and untreated groups. The narrowest scar widths and largest collagen deposition areas were observed in the hiPSC-MSC-Exos group at Days 14 post-wounding, compared to the control and untreated groups. In addition, the collagen fibres in hiPSC-MSC-Exos group showed no loss of periodicity at Day 14. We also observed increased formation of sebaceous glands and hair follicles in hiPSC-MSC-Exos group, relative to the untreated and control groups.

**Figure 4:**
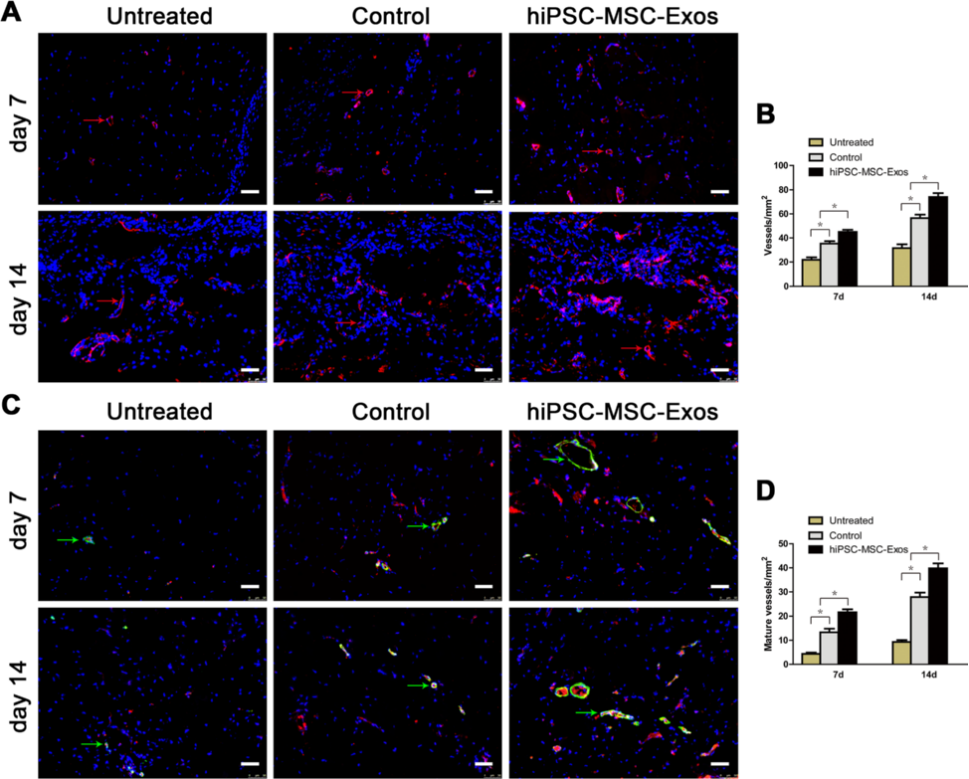
**Immunofluorescence analyses of newly formed vessels and mature vessels. (A)** CD31 (red arrows) Immunofluorescence staining of wound sections treated with PBS, MesenGro hMSC medium, or hiPSC-MSC-Exos at 7 and 14 days post-wounding. Scale bar = 50 μm. **(B)** Enumeration of newly formed vessels in wounds after treatment with PBS, MesenGro hMSC medium, or hiPSC-MSC-Exos at 7 and 14 days post-wounding. **(C)** Immunofluorescent triple staining of wound sections treated with PBS, MesenGro hMSC medium or hiPSC-MSC-Exos at 7 and 14 days post-wounding. Endothelial cells (CD31), smooth muscle cells (α-SMA), and cell nuclei (DAPI) fluoresced with red, green, and blue colours, respectively. Mature vessels (green arrows) were dually positive for CD31 and α-SMA. Scale bar = 50 μm. **(D)** Enumeration of mature vessels in wounds after treatment with PBS, MesenGro hMSC medium, or hiPSC-MSC-Exos at 7 and 14 days post-wounding. *P < 0.05.

Vascularization of newly formed tissues is an essential step in the wound-healing process. Newly formed vessels and mature vessels at wound sites were characterized by CD31 staining (Figure 4A) and co-staining against CD31 and α-SMA (Figure 4C), from which average vessel densities and the number of mature vessels were quantified (Figure 4B, D). These data showed that the number of newly formed vessels and mature vessels both increased along during the healing process for all groups. The hiPSC-MSC-Exos group showed the highest vessel densities and numbers of mature vessels at Day 7 and 14, compared with the control and untreated groups.

### hiPSC-MSC-Exos promote proliferation, migration and collagen, elastin secretion of human fibroblasts in vitro

**Figure 5:**
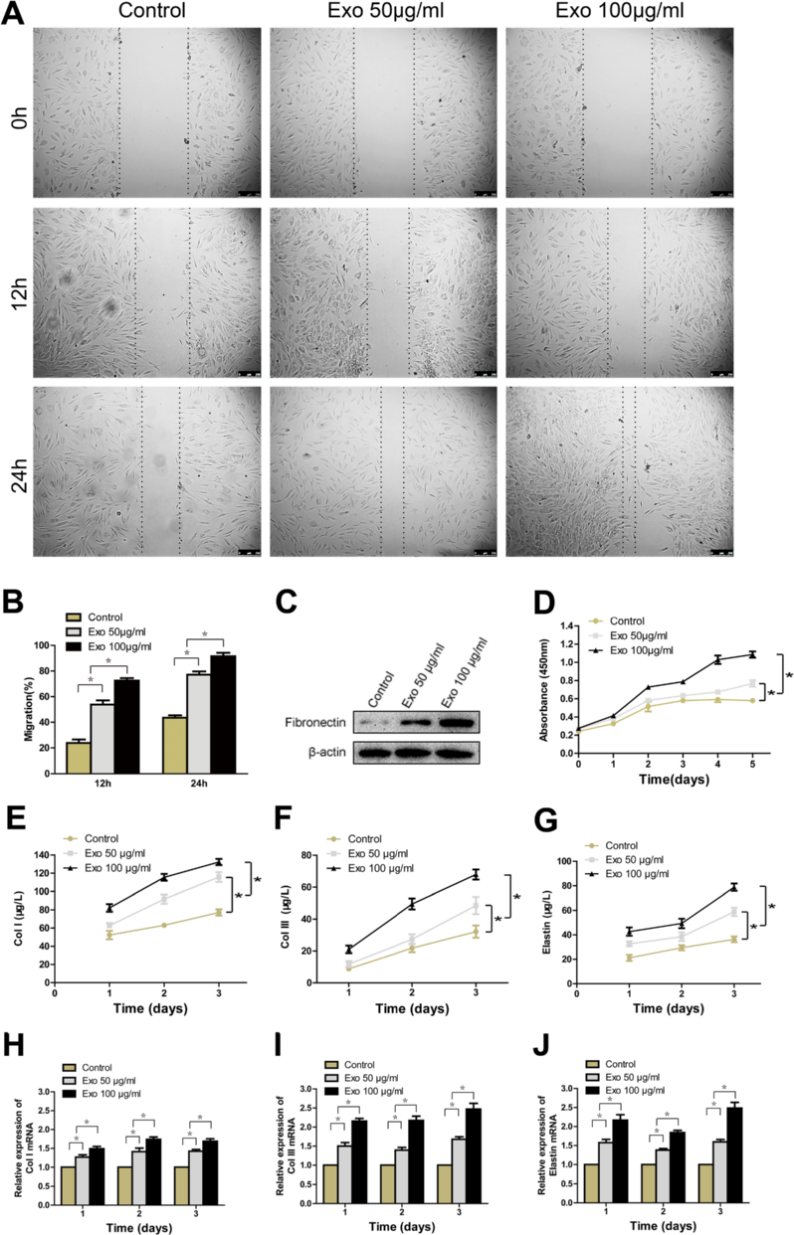
**The effects of exosomes on the proliferation, migration, and collagen, elastin secretion of human fibroblasts.** The light microscopy images **(A)** and migration rates **(B)** of human fibroblasts into scratch sites following growth in MesenGro hMSC medium containing 0, 50, or 100 μg/mL hiPSC-MSC-Exos for 12 or 24 h. Scale bar = 250 μm. **(C)** Fibronectin protein expression of human fibroblasts treated with MesenGro hMSC medium containing 0, 50, or 100 μg/mL hiPSC-MSC-Exos for 24 h. **(D)** Human fibroblasts proliferation after growth in MesenGro hMSC medium containing 0, 50, or 100 μg/mL hiPSC-MSC-Exos was detected with a CCK-8 kit over 5 days. Secretion of Col I **(E)**, III **(F)** and elastin **(G)** by human fibroblasts after growth in MesenGro hMSC medium containing 0, 50, or 100 μg/mL hiPSC-MSC-Exos over 3 days. (G) The Col I **(H)**, III **(I)** and elastin **(J)** mRNA expression of human fibroblasts treated with MesenGro hMSC medium containing 0, 50, or 100 μg/mL hiPSC-MSC-Exos over 3 days. *P < 0.05.

Because we observed enhanced collagen synthesis in wound sites treated with hiPSC-MSC-Exos, we next analysed the underlying mechanism in vitro with primary fibroblasts. Figure 5D demonstrates that fibroblast proliferation increased substantially in the presence of exosomes in a dose-dependent manner.

Compared to control cells, the migration of fibroblasts into scratched areas of monolayers in the presence of 50 μg/mL hiPSC-MSC-Exos increased by 2.3-fold and 1.8-fold after 12 and 24 h, respectively. Similarly, 3.0-fold and 2.1-fold increases in fibroblast migration rates were observed in the presence of 100 μg/mL hiPSC-MSC-Exos after12 and 24 h, respectively, relative to control cells (Figure 5A, B). hiPSC-MSC-Exos can enhance the protein expression of fibronectin in fibroblasts in a dose-dependent manner (Figure 5C).

Figure 5E-G demonstrate that Type I, III collagen and elastin secretion increased in the presence of hiPSC-MSC-Exos at 3 time points in a dose-dependent manner, and the mRNA levels of Type I, III collagen and elastin were also enhanced significantly in a dose-dependent manner (Figure 5H-I). Collectively, these results indicate hiPSC-MSC-Exos can promote proliferation, migration, and collagen secretion of human fibroblasts in vitro.

### hiPSC-MSC-Exos promote proliferation, migration and tube formation of HUVECs in vitro

**Figure 6:**
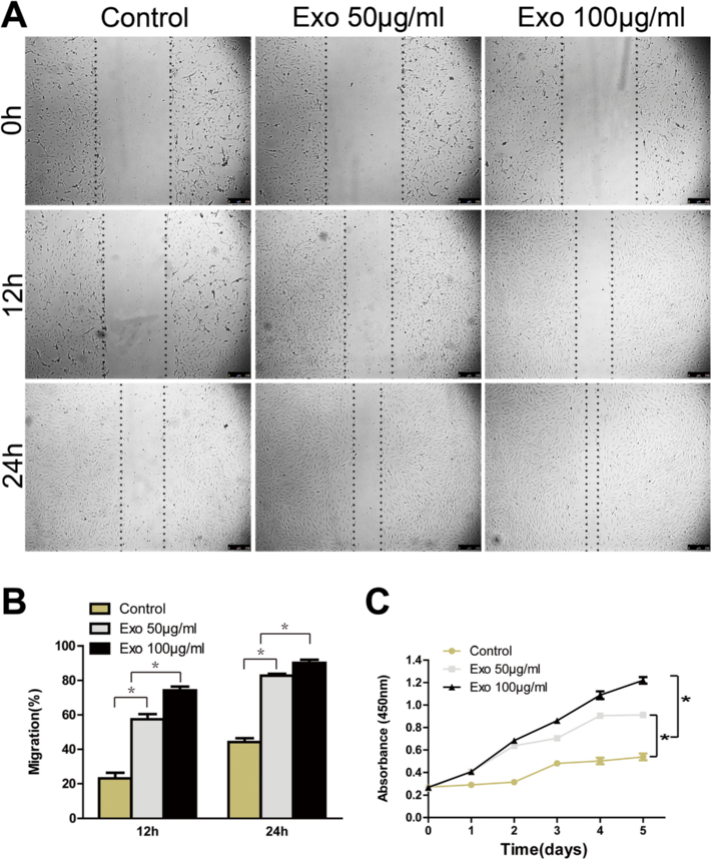
**The effects of exosomes on HUVECs proliferation and migration.** The light microscopy images **(A)** and migration rates **(B)** of HUVECs into the scratched area of monolayers following growth in M200 medium containing 0, 50, or 100 μg/mL hiPSC-MSC-Exos for 12 or 24 h. Scale bar = 250 μm. **(C)** The proliferation of HUVECs grown in M200 containing 0, 50, or 100 μg/mL hiPSC-MSC-Exos was detected over 5 days, using a cell-counting kit.*P < 0.05.

Because enhanced vascularization was also observed in the wound site, we next treated HUVECs with hiPSC-MSC-Exos in vitro to gain insight into the potential mechanism involved. Figure 6C demonstrates that HUVECs proliferation was substantially increased in the presence of exosomes at all time points studied, with the highest proliferation observed in the presence of 100 μg/mL exosomes.

HUVECs migration in the presence of 50 μg/mL exosomes increased by 2.5-fold and 1.9-fold after 12 and 24 h in culture, respectively, and by 3.0-fold and 2.0-fold at the same time points in the presence of 100 μg/mL exosomes (Figure 6A, B).

**Figure 7:**
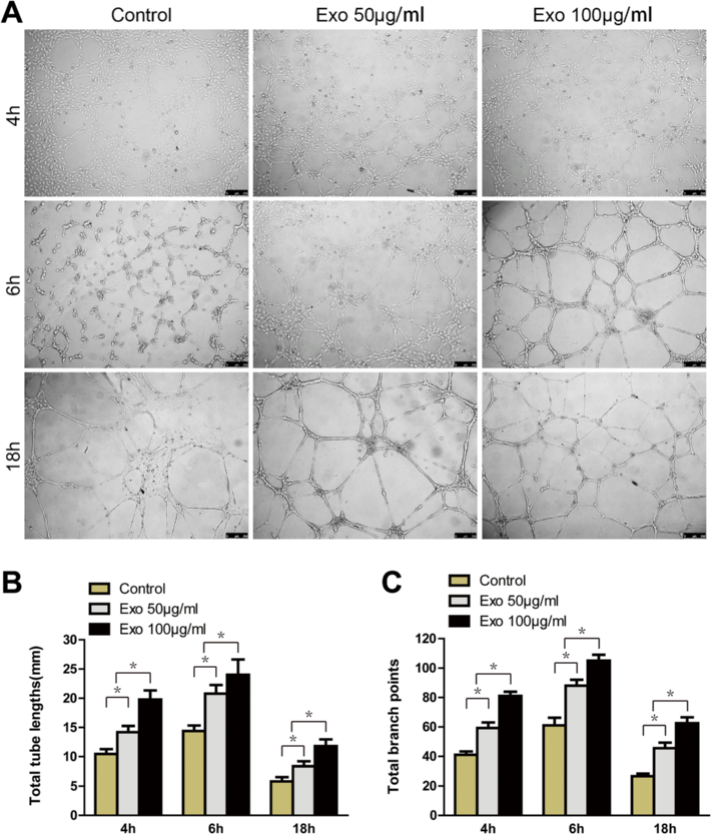
**The effects of exosomes on tube formation by HUVECs. (A)** HUVECs tube formation was studied by growing cells in Matrigel in M200 medium containing 0, 50, or 100 μg/mL hiPSC-MSC-Exos. Scale bar = 250 μm. Total tube lengths **(B)** and branch points **(C)** of HUVECs following growth in M200 medium containing 0, 50, or, 100 μg/mL hiPSC-MSC-Exos for 4, 6, or 18 h. * P < 0.05.

Tubule lengths and branch points were counted after HUVECs began forming capillary tubes. As shown in Figure 7, hiPSC-MSC-Exos markedly enhanced tube formation at 4, 6, and 18 h compared to control cells, with the highest concentration (100 μg/mL) showing the strongest effect. Although the mesh-like structure began dissociating after 18 h in culture, the tubule lengths and degree of branching observed in exosomes-treated cells were enhanced relative to control cells. Collectively, these data indicate that hiPSC-MSC-Exos can promote proliferation, migration, and tube formation of HUVECs in vitro.

## Discussion

In the present study, we investigated whether hiPSC-MSC-Exos can exert therapeutic effects in a cutaneous injury model and explored the underlying mechanisms involved. Our findings demonstrated for the first time that hiPSC-MSC-Exos significantly promote cutaneous wound healing, collagen synthesis, and vascularisation at wound sites in a rat full-thickness skin defect model. Further analysis in vitro indicated that hiPSC-MSC-Exos can promote collagen synthesis in fibroblasts and angiogenesis in HUVECs directly.

It has been suggested that the main underlying mechanism of stem cell transplantation therapy is likely indirect and depends on the paracrine activity of stem cells. Recent studies showed that the exosomes, secreted by cells, strongly contribute to the paracrine effects of stem cells. Thus, we hypothesized that if the benefits of stem cell transplantation therapy are mediated by exosomes, then direct treatment with exosomes may overcome the limitations and risks associated with stem cell transplantation therapy. Previous studies also demonstrated that human MSC-derived exosomes do not contain MHC class I or II proteins and thus, their application to non-immune-compatible animals does not induce overt immune reactions [36-38], making them suitable for use in allogeneic or xenogeneic recipients. iPSC-MSCs have emerged as an alternative resource for stem cell transplantation therapy. Our study demonstrated that hiPSC-MSCs display MSC-like properties and multipotency. Previous studies have shown that iPSC-MSCs may be produced abundantly, have a strong capacity for self-renewal, and can attenuate tissue ischemia for an improved therapeutic effect compared to adult bone marrow mesenchymal stem cells [9]. Thus, in the present study, exosomes derived from hiPSC-MSCs were first adopted to evaluate their effects in cutaneous wound healing in a rat full-thickness skin defect model.

It was clearly observed that the application of exosomes greatly increased re-epithelization and collagen deposition at wound sites, the nascent collagen fibrils also showed no loss of periodicity. Meanwhile, exosomes not only promoted the generation of newly formed vessels, but also accelerated their maturation at wound sites. The encouraging results suggested that hiPSC-MSC-Exos have similar reparative properties as MSCs in tissue repair. hiPSC-MSC-Exos appear to be a superior candidate for treating cutaneous wound healing, that might overcome the obstacles and risks associated with stem cell transplantation therapy.

The formation of granulation tissues is a major determinant in wound healing, providing a scaffold for the assembly of neighbouring cells at wound margins, contributing to wound closure. Fibroblasts are the main cell types comprising granulation tissue. Fibroblasts are present in the dermis and proliferate rapidly and migrate to wound sites [39,40], where they can secrete Type I and III collagens and elastin, which are the central components of the extracellular matrix. Studies have demonstrated that MSCs can promote the proliferation, migration, and collagen secretion of fibroblasts through a paracrine mechanism [41]. We showed that hiPSC-MSC-Exos stimulated the proliferation and migration of human dermal fibroblasts in a dose-dependent manner in vitro, and that Type I, III collagen and elastin secretion was increased with increasing hiPSC-MSC-Exos concentrations. Extracellular matrix (ECM) is comprised of various substances that promote the migration of fibroblasts (fibronectin) and provide tissue strength and resiliency (collagen and elastin). The effects of hiPSC-MSC-Exos on fibroblasts migration were confirmed in cell responses level by western blot measurement of fibronectin protein levels. These factors may contribute to wound contraction and collagen maturation during cutaneous wound healing. Angiogenesis is also known to play a critical role in the cutaneous wound-healing process and is required for granulation tissue formation. Previous studies showed that BMSCs-derived exosomes can significantly increase the proliferation, migration, and tube formation ability of HUVECs [42] and that exosomes are potentially useful in treating certain ischemic diseases. However, no reports have thus far described the application of hiPSC-MSC-Exos incutaneous wound healing. Our in vitro experimental results confirmed that hiPSC-MSC-Exos can increase proliferation, migration, and tube formation of HUVECs in a dose-dependent manner. These data indicate that the formation of nascent and mature vessels during cutaneous wound healing may be due to the pro-angiogenic effect of hiPSC-MSC-Exos.

## Conclusion

We demonstrated that hiPSC-MSC-Exos exert beneficial effects on granulation tissue formation and angiogenesis, which are two critical phases of the wound-healing process, and that hiPSC-MSC-Exos facilitated a significant therapeutic effect during cutaneous wound healing. Our findings suggest that hiPSC-MSC-Exos may be used as therapeutic tools in cutaneous wound healing.
